# Estimating peer density effects on oral health for community-based older adults

**DOI:** 10.1186/s12903-017-0456-4

**Published:** 2017-12-29

**Authors:** Bibhas Chakraborty, Michael J. Widener, Sedigheh Mirzaei Salehabadi, Mary E. Northridge, Susan S. Kum, Zhu Jin, Carol Kunzel, Harvey D. Palmer, Sara S. Metcalf

**Affiliations:** 1 Center for Quantitative Medicine, Duke-National University of Singapore (Duke-NUS) Medical School, Singapore, 169857 Singapore; 2 Department of Geography and Planning, University of Toronto, Toronto, ON M5S 1S8 Canada; 3 Department of Epidemiology & Health Promotion, New York University College of Dentistry, New York, NY 10010 USA; 4 Department of Sociomedical Sciences, Columbia University Mailman School of Public Health, New York, NY 10032 USA; 5 Department of Geography, The State University of New York at Buffalo, Buffalo, NY 14261 USA; 6 Section of Population Oral Health, Columbia University College of Dental Medicine, New York, NY 10032 USA; 7 Department of Political Science, The State University of New York at Buffalo, Buffalo, NY 14260 USA

**Keywords:** Oral health equity, Racial/ethnic minorities, Older adults, Community-based oral health care, Social support, Social networks, Knowledge diffusion, Kernel density estimation, Logistic regression, Multiple imputation

## Abstract

**Background:**

As part of a long-standing line of research regarding how peer density affects health, researchers have sought to understand the multifaceted ways that the density of contemporaries living and interacting in proximity to one another influence social networks and knowledge diffusion, and subsequently health and well-being. This study examined peer density effects on oral health for racial/ethnic minority older adults living in northern Manhattan and the Bronx, New York, NY.

**Methods:**

Peer age-group density was estimated by smoothing US Census data with 4 kernel bandwidths ranging from 0.25 to 1.50 mile. Logistic regression models were developed using these spatial measures and data from the *ElderSmile* oral and general health screening program that serves predominantly racial/ethnic minority older adults at community centers in northern Manhattan and the Bronx. The oral health outcomes modeled as dependent variables were ordinal dentition status and binary self-rated oral health. After construction of kernel density surfaces and multiple imputation of missing data, logistic regression analyses were performed to estimate the effects of peer density and other sociodemographic characteristics on the oral health outcomes of dentition status and self-rated oral health.

**Results:**

Overall, higher peer density was associated with better oral health for older adults when estimated using smaller bandwidths (0.25 and 0.50 mile). That is, statistically significant relationships (*p* < 0.01) between peer density and improved dentition status were found when peer density was measured assuming a more local social network. As with dentition status, a positive significant association was found between peer density and fair or better self-rated oral health when peer density was measured assuming a more local social network.

**Conclusions:**

This study provides novel evidence that the oral health of community-based older adults is affected by peer density in an urban environment. To the extent that peer density signifies the potential for social interaction and support, the positive significant effects of peer density on improved oral health point to the importance of place in promoting social interaction as a component of healthy aging. Proximity to peers and their knowledge of local resources may facilitate utilization of community-based oral health care.

## Background

There is currently unprecedented growth in the number and proportion of older adults in the United States, creating challenges in the delivery of oral health care services, especially for disadvantaged members of the population [1]. Indeed, over the next 25 years, the joint effect of longer life spans and the aging of the baby boom generation is expected to double the population of Americans aged 50 years and older [2]. As a result of this demographic shift, increased public health attention is being focused on place-based strategies that integrate services and support older populations in the communities where they live [3, 4]. A first priority of health practitioners is to better ensure the oral health and well-being of older adults across the socioeconomic spectrum [5]; a corollary is to target disadvantaged subpopulations with supportive public health programs and policies [6]. A second priority is to shift oral health care spending to fund preventive activities in familiar, community-based settings rather than restorative treatments [6–8].

Accordingly, one strategy is to take a holistic view of health and integrate oral and general health screening at community sites where older adults gather [7, 8]. Dental disease is largely preventable [6], and oral health is vital to general health and well-being [6, 9]. Moreover, oral health care providers may detect chronic health conditions such as diabetes [10], as the mouth reflects overall health throughout the life course [6]. Disadvantaged older adults, including racial/ethnic minority populations who live in impoverished urban neighborhoods, may require invasive dental procedures (periodontal therapy and tooth extraction) that increase the incidence of ischemic stroke and myocardial infarctions, and which would likely have been avoidable with prevention and early treatment [11].

Beyond the direct health advantages of a preventive approach, oral health may reinforce interactions with others, thereby encouraging social contact and constructive behaviors such as participating in community-based events [12]. Recent research found evidence of a positive relationship between higher levels of self-reported oral health and the peer density of older adults, defined as the spatial concentration of similarly aged residents [13]. This relationship between the social context (i.e., a higher concentration of peers with whom to interact) and oral health suggests that there is a positive feedback loop, with social contact leading to knowledge networks, resulting in improved dental hygiene and oral health care seeking behavior [14].

These latter findings on oral health and health care [13, 14] contribute to a long-standing line of research regarding how peer density affects health [15]. In particular, researchers have sought to understand the multifaceted ways that the density of peers living and interacting in proximity to one another influence social networks and knowledge diffusion, and subsequently health and well-being [16].

Relationships between peer density and health are undoubtedly complex. Peer density may indicate the potential for social interaction and support to induce positive effects on health [17], but high levels of peer density have also been found to be related to low levels of mental health [18]. Research on recent immigrants from the same country of origin found a nonlinear relationship between peer density and health, in which both low and high levels of peer density are associated with higher rates of low birth weight babies, whereas medium levels of peer density are associated with lower rates of low birth weight babies [19]. Such results point to a lack of social support for recent immigrants in low density areas, and overcrowding for recent immigrants in high density areas [19]. These mixed effects underscore the importance of understanding the specific populations, spatial context, and outcomes of interest in interpreting relationships between peer density and health. The present study contributes to the literature on the relationship between peer density and the oral health of older adults in an urban context by examining effects on dentition status and contrasting these findings with those obtained for self-rated oral health.

## Methods

This study is cross-sectional in design. The analyses of peer density effects on dentition status and self-rated oral health are based upon data obtained from both a community-based clinical screening program and the US Census [20]. A methodological advance of this study is the use of multiple imputation of explanatory variables as a strategy for dealing with missing data in making statistical inferences about the outcomes of interest [21].

**Fig. 1:**
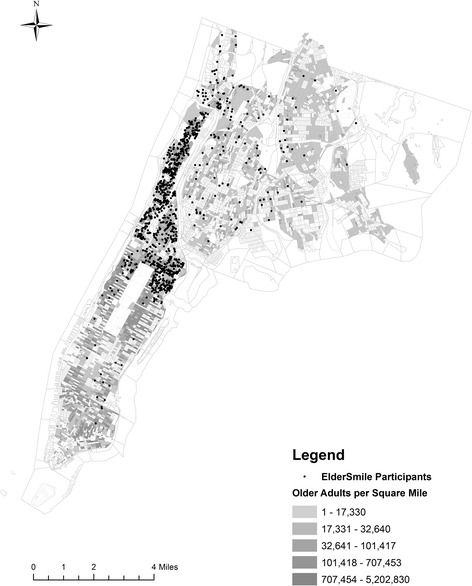
Point locations of residences of *ElderSmile* participants who are aged 50 years and older from Manhattan and the Bronx, New York City, NY, 2006-2013 (*n* = 1822), overlaid on top of Census block-level density of the total population aged 50 years and older in 2010

In particular, the sociodemographic, health, health care, and geographic data for this research are derived from participants in the *ElderSmile* program [7, 8, 22–26]. Briefly, the *ElderSmile* program is an initiative of the Columbia University College of Dental Medicine and its partners. Provided services include oral and general health education and preventive screenings at locations where older adults gather, such as senior centers and senior housing facilities, within the communities of northern Manhattan and the Bronx, New York, NY [7, 8, 22–26]. No one who sought services as part of this initiative was turned away. Analyses were conducted with data from *ElderSmile* participants aged 50 years and older whose residences were geocoded to locations in northern Manhattan and the Bronx (see Fig. 1).

A total of 1822 participant records were available for analysis for the period from 2006 to 2013. Note that a subset of this sample was previously used to examine peer density effects on self-rated oral health [13]. The present study includes 3 additional years of *ElderSmile* program data and updated US Census population data [20].

### Kernel density estimation

Population data for adults aged 50 years and older were obtained from the 2010 US Decennial Census [20] and assigned to the centroids of the respective Census blocks (as mapped in Fig. 1). Kernel density estimation was used to produce smoothed peer density surfaces based on the older adult population associated with the centroid of each Census block. Kernel density estimate (KDE) values were then assigned to each participant record in the dataset based upon their geocoded residential location.

The process of kernel density estimation produces a smoothed density surface based upon the distribution of observed values at different locations (i.e., the number of adults aged 50 years and older associated with Census block centroids). In essence, kernel estimators smooth out the contribution of each observed data point over its local neighborhood. The contribution of the *i* ^th^ data point *x*_*i*_ (*i* = 1, …, *n*) to the estimate at a given location *x* depends upon how far apart *x*_*i*_ and *x* are geographically. The extent of this contribution is dependent upon the shape of the kernel function adopted and its bandwidth. If the kernel function is denoted as *K*(•) and its bandwidth by *h*, the general KDE function at any point *x* is calculated as:

$$ \widehat{f}(x)=\frac{1}{nh}\sum \limits_{i=1}^n\ K\left(\frac{x-{x}_i}{h}\right) $$

where *x*_1_, *x*_2_, …, *x*_*n*_ are *n* observed points [27]. The kernel function *K*(•) is non-negative and integrates to 1. Here, a quadratic form is used for *K*(•), such that *K*(*u*) = ¾(1-*u* ^2^) if |*u*| ≤ 1 and *K*(*u*) = 0 if |*u*| > 1. Since *u* = (*x-x*_*i*_)/*h*, this condition ensures that the quadratic form of the kernel function is applied where the distance between the given point *x* and the observed point *x*_*i*_ is no greater than the bandwidth *h*, thus accounting only for the observed points that are within the search window from the given location. The peer density (KDE) estimated by $\widehat{f}(x)$ in this study has units of adults aged 50 years and older per square mile.

**Fig. 2:**
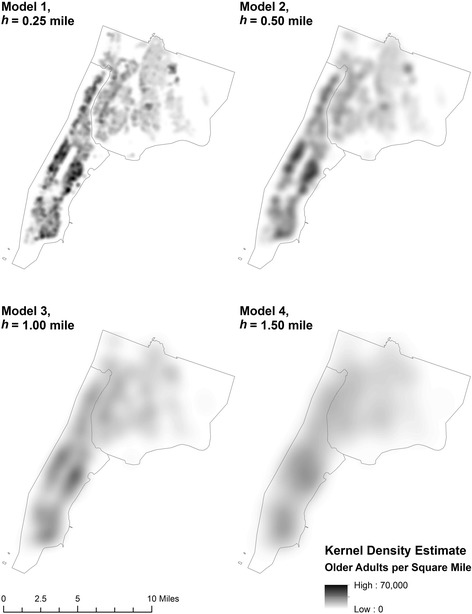
Kernel density estimation surfaces generated for the population of older adults (aged 50 years and older) in northern Manhattan and the Bronx, New York, NY using different bandwidths (*h*) to define 4 spatial models used in logistic regression analyses of oral health outcomes. Kernel density estimate (KDE) values have units of the number of older adults per square mile

The bandwidth parameter (*h*) of the kernel function determines the size of the search window in estimating kernel density. Because of its importance in estimating the kernel density surface, 4 different values of bandwidth were used in this study to calculate the density surfaces, i.e., *h* = 0.25, 0.50, 1.00, and 1.50 miles. These bandwidth values were selected to encompass areas within walking distance from the residence of a participant, such as senior centers and other third places [8] where community-based older adults are likely to socialize [13]. While we expect a bandwidth of 0.25 or 0.50 mile to better represent the typical socializing radius or ambit [28] of older adults in urban areas, larger bandwidths were also employed to assess model robustness, i.e., how sensitive our peer density findings are to this measurement assumption. Figure 2 compares the kernel density surfaces for each of these 4 bandwidth values that distinguish the 4 spatial models used in the subsequent logistic regression analyses of effects on oral health outcomes.

As illustrated in Fig. 2, larger bandwidths generate smoother density estimates over space, as more distant observations are included in the calculation and data observed farther away thereby exert a greater influence on the estimation of kernel density for a given resident. In contrast, smaller bandwidths produce more local variation and distinct clusters. For instance, the kernel density surface created for Model 2 at *h* = 0.50 mile smooths over the small clusters that appear in Model 1 as the smallest bandwidth surface, but retains greater spatial variation in density than the surfaces for Models 3 and 4 using larger bandwidths.

### Variables

Logistic regression analyses were performed to examine 2 oral health outcomes, dentition status and self-rated oral health, using 4 spatial models that include the KDE measure from different bandwidth versions of peer density as the main determinant of interest. A summary of the variables used for the generalized adjacent-categories logistic regression of ordinal dentition status and for the standard logistic regression of binary self-rated oral health is presented in Table 1.

**Table 1:** Variables used in generalized adjacent-categories logistic regression of dentition status and in standard logistic regression of self-rated oral health: *ElderSmile* program, New York, NY, 2006-2013 (*n* = 1822)

Variables	Missing	Proportion				Standard			
	Values	=0	=1	=2	=3	Mean	Deviation	Min.	Max.
*Outcome Variables*									
Dentition status(0 = functional; 1 = limited;2 = edentulous)	18.2%	35.7%	44.6%	19.7%	–	–	–	0	2
Self-rated oral health (0 = poor;1 = fair or better)	15.4%	22.2%	77.8%	–	–	–	–	0	1
*Explanatory Variables*									
Gender(0 = male; 1 = female)	0.933%	27.3%	72.7%	–	–	–	–	0	1
Race/ethnicity(0 = Hispanic;1 = White; 2 = Black;3 = Other)	4.83%	57.2%	11.2%	28.2%	3.46%	–	–	0	3
Agein years	2.14%	–	–	–	–	73.48	10.07	50	105
Smoking status(0 = current; 1 = former; 2 = never)	24.8%	11.6%	30.3%	58.1%	–	–	–	0	2
Medicaid dental insurance	13.8%	48.3%	51.7%	–	–	–	–	0	1
Private dental insurance	13.7%	93.3%	6.74%	–	–	–	–	0	1
Education (0 = primary; 1 = high school; 2 = college)	11.5%	37.8%	35.0%	26.9%	–	–	–	0	2
Peer density (KDE) for Model 1,*h* = 0.25 mile	–	–	–	–	–	27,320	9290	3277	59,370
Peer density (KDE) for Model 2,*h* = 0.50 mile	–	–	–	–	–	22,280	6274	3081	53,190
Peer density (KDE) for Model 3,*h* = 1.00 mile	–	–	–	–	–	17,690	3789	3796	31,820
Peer density (KDE) for Model 4,*h* = 1.50 mile	–	–	–	–	–	15,740	3010	2492	27,130

*Note: KDE* kernel density estimate, in units of older adults (aged 50 years and older) per square mile

The first oral health outcome of interest was dentition status, derived from the presence or absence of teeth as measured by an *ElderSmile* dentist based upon a complete dentition of 28 teeth. Third molars were excluded from the analyses because they are often missing for reasons other than dental caries or other oral diseases. Being edentulous was defined as having no natural permanent teeth in the mouth or 28 missing teeth [26, 29]. Because having 20 teeth is considered necessary for functional dentition [6, 26, 30], participants with 0 to 8 missing teeth were considered to have functional dentition, and participants with 9 to 27 missing teeth were considered to have limited functional capacity. Therefore, for this study, dentition status was coded as an ordinal variable, where 0 = functional dentition, 1 = limited functional capacity, and 2 = edentulous.

The second oral health outcome of interest was self-rated oral health. On the program intake form, *ElderSmile* participants rated their oral health as excellent, good, fair, or poor [25]. A weighted kappa statistic demonstrated a significant level of agreement between the self-rated oral health of *ElderSmile* participants and dentist-rated oral hygiene; in particular, untreated dental caries and severe periodontal inflammation were significantly related to poor self-rated oral health [13, 25]. This appears to be an important cutpoint (i.e., at least fair versus poor) in this community-based sample of older adults based upon the stratified analyses and the goodness of fit of the logistic regression models. It may be that what matters most for underserved older adults in rating their oral health is pain related to untreated disease, rather than aesthetics or oral hygiene, and thus there appears to be a lower cutpoint in the ElderSmile program sample than that found in other population-based samples (i.e., excellent or good versus fair or poor). The 4-category self-rated oral health variable was thus recoded as a binary variable for the analyses, where a value of 0 represents poor self-rated oral health and a value of 1 represents at least fair or better self-rated oral health [13, 25].

In addition to the KDEs described above as the spatially-derived determinants of interest, all other covariates used in the analyses were derived from the *ElderSmile* program intake form. Peer density (KDE) and age (in years) were treated as continuous covariates. Of the other covariates, 3 were coded as binary: gender (0 = male, 1 = female); whether or not they have Medicaid coverage (1 = has Medicaid coverage, 0 = does not have Medicaid coverage); and whether or not they have private dental insurance (1 = has private dental insurance, 0 = does not have private dental insurance). The 3 remaining covariates were treated as categorical: race/ethnicity (0 = Hispanic, 1 = non-Hispanic White, 2 = non-Hispanic Black, 3 = Other); education level (0 = primary school, 1 = high school, 2 = college); and smoking status (0 = current smoker, 1 = former smoker, 2 = never smoked). The distribution of racial/ethnic identities among *ElderSmile* participants reflects the predominantly Hispanic (largely Dominican and Puerto Rican) and African American communities served by the program in the neighborhoods of northern Manhattan and the Bronx. Because most participants (57.2%) identified as Hispanic, this group was selected as the reference category in specifying the effects of race/ethnicity in the models. Of the 3 education levels, the greatest proportion of participants (37.8%) indicated primary school as their highest level of educational attainment.

### Multiple imputation of missing data

When analyzing datasets with partial missing information, researchers either ignore all of the available information for participants with missing values (which results in loss of information and introduces bias into the findings) or elect to use all of the available information at hand by first imputing the missing data and then analyzing the imputed versions of the dataset. Percentages of missing values for the variables used in the logistic regression models are listed in Table 1 above. Note the high rates of missing data for certain variables such as smoking status (24.8%). Imputation of these missing values (rather than exclusion of these records) ensures that the sample of participants analyzed in this study is representative of the population of participants in the *ElderSmile* program. Missing values were imputed using a Bayesian statistical technique known as multiple imputation [21, 31], which has been used in other health research [32, 33].

A foundational assumption for multiple imputation is that data are missing at random:

$$ f\left(M,\mid, {Y}_{obs},{Y}_{mis}\right)=f\left(M|{Y}_{obs}\right) $$

where *M* denotes that one observation is missing, *Y*_*obs*_ denotes the observed values, and *Y*_*mis*_ denotes the missing values in the target data set. Under this assumption, the probability that an observation is missing depends on the whole population only through the observed value *Y*_*obs*_. It may be derived that any inference on *θ* depends only on the observed data likelihood:

$$ L\left(\theta |{Y}_{obs}\right)\propto L\left({Y}_{obs}|\theta \right) $$

where *θ* is the potential parameter for the data distribution [34]. By Bayesian inference, the posterior distribution of *θ* depends on:

$$ P\left(\theta |{Y}_{obs}\right)\propto L\left(\theta |{Y}_{obs}\right)\pi \left(\theta \right) $$

where *π* is the prior distribution of *θ* [35]. Multiple imputation draws values from the posterior predictive distribution of *Y*_*mis*_, *P*(*Y*_*mis*_| *Y*_*obs*_) instead of from *Y*_*mis*_ [36]. Denoting *P*(*Y*_*mis*_| *Y*_*obs*_, *θ*) as the conditional predictive distribution of *Y*_*mis*_, the posterior predictive distribution of *Y*_*mis*_ may be written as:

$$ P\left({Y}_{mis}|{Y}_{obs}\right)=\int P\left({Y}_{mis}|{Y}_{obs},\theta \right)P\left(\theta |{Y}_{obs}\right) d\theta . $$

Different models for multiple imputation are appropriate for different variable types. For a continuous target variable (e.g., participant age in years), predictive mean matching is used to impute missing values. In this model, the 5 most highly correlated variables are used as predictors, denoted *X*_1_, …, *X*_5_. The linear model:

$$ Y={\beta}_0+{\upbeta}_1{X}_1+\dots +{\beta}_5{X}_5+\epsilon $$

is thus fitted using observations with observed values for the variable *Y* and its covariates *X*_1_, …, *X*_5_,where *ϵ* ∼ *N*(0, *σ* ^2^). By specifying the prior distribution of the parameters ***β*** = (*β*_0_, …, *β*_5_)^*T*^and *σ*, ***β*** ^∗^ and *σ* ^∗^may be drawn from their posterior distributions. Hence, the predicted value for each observed *y*_*l*_ may be obtained by:

$$ {\widehat{\mu}}_l={\widehat{\beta}}_0+{\widehat{\beta}}_1{x}_{1l}+\dots +{\widehat{\beta}}_5{x}_{5l} $$

Then the imputation value of a missing *y*_*j*_ will be:

$$ {\widehat{y}}_j={y}_k $$

where ${\left({\widehat{\mu}}_j-{\widehat{\mu}}_k\right)}^2\le {\left({\widehat{\mu}}_j-{\widehat{\mu}}_l\right)}^2$ for all observed *l*, ${\widehat{\mu}}_j$ is the predicted mean of *Y* and *y*_*k*_ is the observed value of *Y* for the *k*th observation [37].

Imputation using logistic regression is appropriate if the target variable *Y* is binary (e.g., gender). Define *p* as the probability that *Y* = 1. Consider the logistic regression model:

$$ logit\ p={\beta}_0+{\beta}_1{X}_1+\dots +{\beta}_5{X}_5 $$

where *X*_1_, …, *X*_5_ are the top 5 highly correlated variables for target variable *Y* and $logit\ p=\ln \left(\frac{p}{1-p}\right)$. New parameters${\boldsymbol{\beta}}^{\ast }={\left({\beta}_0^{\ast },\dots, {\beta}_5^{\ast}\right)}^T$ are drawn from the posterior distribution of ***β***. Then, for an observation with missing *Y*_*l*_ and covariates *x*_1*l*_, …, *x*_5*l*_, the expected probability that *Y*_*l*_ = 1 is:

$$ {\widehat{p}}_l=\frac{\exp \left({\widehat{\mu}}_l\right)}{1+\exp \left({\widehat{\mu}}_l\right)} $$

where ${\widehat{\mu}}_l={\beta}_0^{\ast }+{\beta}_1^{\ast }{x}_{1l}+\dots +{\beta}_5^{\ast }{x}_{5l}$. The imputed value is then drawn based on the estimated probability ${\widehat{p}}_l$ [21].

Imputation using polytomous regression is needed if the target variable *Y* is categorical or ordinal, e.g., the 3-category measure of smoking status. Define probability *p*_0_, *p*_1_ and *p*_2_ as follows:

$$ {\displaystyle \begin{array}{c}{p}_0=P\left(Y=0\right)\ \\ {}{p}_1=P\left(Y=1\right)\\ {}{p}_2=P\left(Y=2\right)=1-{p}_0-{p}_1\ \end{array}} $$

Next, consider the multinomial logit model:

$$ {\displaystyle \begin{array}{c}{p}_0=\frac{\exp \left({a}_0+{\boldsymbol{b}}_0\boldsymbol{X}\right)}{1+\exp \left({a}_0+{\boldsymbol{b}}_0\boldsymbol{X}\right)+\exp \left({a}_1+{\boldsymbol{b}}_1\boldsymbol{X}\right)}\ \\ {}{p}_1=\frac{\exp \left({a}_1+{\boldsymbol{b}}_1\boldsymbol{X}\right)}{1+\exp \left({a}_0+{\boldsymbol{b}}_0\boldsymbol{X}\right)+\exp \left({a}_1+{\boldsymbol{b}}_1\boldsymbol{X}\right)}\ \\ {}{p}_2=\frac{1}{1+\exp \left({a}_0+{\boldsymbol{b}}_0\boldsymbol{X}\right)+\exp \left({a}_1+{\boldsymbol{b}}_1\boldsymbol{X}\right)}\ \end{array}} $$

where ***X*** = (*X*_1_, …, *X*_5_)^*T*^ is the vector containing the 5 most highly correlated variables for *Y* as predictors. The estimated parameters ${a}_0^{\ast },{a}_1^{\ast },{\boldsymbol{b}}_0^{\ast },{\boldsymbol{b}}_1^{\ast }$ are drawn from the posterior distributions. These parametric estimates are then used to calculate the estimated probabilities of each possible outcome (i.e., 0 = current smoker, 1 = former smoker, and 2 = never smoked) for each missing *Y*. The imputed value for each missing *Y* is then randomly drawn based upon these estimated probabilities [38].

The approach to multiple imputation employed in this study involved constructing *m* = 10 different datasets by estimating each missing observation *m* times, performing data analysis on each of the imputed datasets, and combining the results from the analysis of each dataset. The combined results, derived from the separate analyses of the 10 multiple-imputed datasets, were then used as a basis for statistical inference.

In this study, multiple imputation was performed for explanatory variables, whereas complete cases were used for the outcome variables of dentition status and self-rated oral health. This approach was adopted due to the nature of the outcome variables and to address the potential concern among certain researchers that imputation of missing outcomes produces overly optimistic standard errors. For self-rated oral health, missingness is likely a distinct outcome (i.e., someone who actually does not know how to assess their health status) rather than an unobserved one (i.e., someone who has an assessment in mind but does not want to report it). For dentition status, the rationale against imputation rests on the stronger assumption that the missingness is likely to be completely at random given that it occurs due to participants opting out of a dental examination rather than survey item nonresponse (which is the nature of missingness for the explanatory variables). Given the statistical concern, though, that listwise deletion of cases due to missingness can introduce estimation bias [39], sensitivity analyses were performed to assess robustness relative to analyses in which all variables, including the 2 outcome variables, were imputed and those in which only complete cases were included (without imputation). Overall, the results of these auxiliary analyses (available from the authors upon request) are qualitatively similar in terms of the effects of peer density and other covariates.

### Analysis

After construction of the kernel density surfaces and multiple imputation of missing data for the explanatory variables, logistic regression analyses were performed to estimate the effects of peer density and other sociodemographic characteristics on the oral health outcomes of dentition status and self-rated oral health. For each of these 2 outcome variables, a set of 4 logistic regression models was estimated with specifications that differ in the measure of peer density included, where the KDE surface was constructed using 4 distinct bandwidth parameter *h* values as follows: 0.25 mile (Model 1), 0.50 mile (Model 2), 1.00 mile (Model 3), and 1.50 miles (Model 4). These 4 spatial models provide alternative estimates of the effect of peer density based on different characterizations of a participant’s social network, with Model 1 including the most local measure and Model 4 including the most dispersed measure (see Fig. 2).

Generalized adjacent-categories logistic regression (GACLR) models [40] were developed to estimate the effects of peer density and other covariates on dentition status, as indicated by 3 ordered categories (0 = functional dentition or 0-8 missing teeth; 1 = limited functional capacity or 9-27 missing teeth; and 2 = edentulous or 28 missing teeth) [6, 26, 30]. The general form of the GACLR model is:

$$ \ln \left[\frac{P\left(Y=j+1\right)}{P\left(Y=j\right)}\right]={\beta}_{0j}+{\beta}_{1j}{X}_1+{\beta}_{2j}{X}_2+\cdots +{\beta}_{qj}{X}_q $$

where *X*_*1*_ *, X*_*2*_ *, …, X*_*q*_ are the set of covariates used in the model for comparing the odds of the category (*j* + 1) relative to its adjacent category *j* of the ordinal outcome *Y*; the parameters *β*_*1j*_ *, β*_*2j*_ *, …, β*_*qj*_ are the corresponding regression coefficients, and *β*_*0j*_ denotes the intercept. As in standard logistic regression, the exponential transform of the regression coefficient, exp.(*β*_*1j*_), denotes the odds ratio (OR) of the covariate *X*_*1*_ for comparing the category *(j* + 1*)* relative to its adjacent category *j* of the ordinal outcome *Y*, and so on*.* The GACLR model was chosen over other alternatives for analyzing ordinal outcome variables for 2 reasons. First, the GACLR model does not assume the restrictive proportional odds assumption [40], thereby allowing for parametric differences in the effects of the predictions on the relative probabilities of different adjacent-outcome pairs (i.e., different for limited functional capacity relative to functional dentition than for edentulous relative to limited functional capacity). Second, given the importance of being able to calculate incidence rates, the GACLR model is easier to interpret since it directly estimates the effects of the predictors on the OR of each adjacent-outcome pair rather than requiring these to be derived via nonlinear functions that necessitate estimation of standard errors using simulation or resampling methods.

Standard logistic regression models were developed for self-rated oral health as the second oral health outcome of interest, using the binary outcome of poor vs. fair or better self-rated oral health, with the same set of 4 spatial models and covariates as considered in case of the ordinal dentition status outcome. All analyses were conducted in the statistical software R version 3.1.2 (https://cran.r-project.org/) using the package vector generalized linear and additive models (VGAM). All appropriate institutional review board and Health Insurance Portability and Accountability Act safeguards were followed.

## Results

The results from the GACLR analysis corresponding to the 4 spatial models described above are presented in Table 2 for the dentition outcomes of limited functional capacity (Y = 1) relative to functional dentition (Y = 0) and for edentulous (Y = 2) relative to limited functional capacity (Y = 1).

**Table 2:** Odds ratios (OR) and 95% confidence intervals (CI) for limited functional capacity (Y = 1) relative to functional dentition (Y = 0) and for edentulous (Y = 2) relative to limited functional capacity (Y = 1) resulting from generalized adjacent-categories logistic regression with 4 spatial models defined by kernel bandwidth *h*: *ElderSmile* program, New York, NY, 2006-2013 (*n* = 1490 complete cases for dentition status)

Variables	Model 1,*h* = 0.25 mile	Model 2,*h* = 0.5 mile	Model 3,*h* = 1.0 mile	Model 4,*h* = 1.5 mile
	OR (95% CI)	OR (95% CI)	OR (95% CI)	OR (95% CI)
Limited Functional Capacity (Y = 1) vs. Functional Dentition (Y = 0)				
Female	1.208***(1.143, 1.277)	1.217***(1.152, 1.285)	1.217***(1.152, 1.285)	1.213***(1.149, 1.285)
Non-Hispanic White	0.320***(0.277, 0.370)	0.329***(0.284, 0.380)	0.347***(0.300, 0.401)	0.353***(0.305, 0.380)
Non-Hispanic Black	1.090***(1.030, 1.153)	1.143***(1.080, 1.209)	1.181***(1.115, 1.250)	1.166***(1.099, 1.212)
Other non-Hispanic race/ethnicity	0.593***(0.463, 0.760)	0.584***(0.459, 0.743)	0.595***(0.467, 0.758)	0.592***(0.464, 0.744)
Age in years	1.0379***(1.0347, 1.0412)	1.0372***(1.0339, 1.0404)	1.0369***(1.0336, 1.0402)	1.0370***(1.0337, 1.0405)
Former smoker	0.878(0.654, 1.179)	0.878(0.652, 1.183)	0.892(0.662, 1.202)	0.894(0.664, 1.182)
Never smoked	0.565***(0.426, 0.749)	0.563***(0.425, 0.748)	0.567***(0.427, 0.751)	0.570***(0.430, 0.747)
Medicaid dental insurance	1.450***(1.145, 1.836)	1.460***(1.148, 1.856)	1.456***(1.146, 1.850)	1.448***(1.139, 1.856)
Private dental insurance	0.874(0.715, 1.069)	0.899(0.736, 1.098)	0.922(0.757, 1.124)	0.926(0.759, 1.096)
High school education	1.064(0.957, 1.183)	1.078(0.969, 1.200)	1.096*(0.987, 1.219)	1.099*(0.990, 1.197)
Collegeeducation	0.903(0.773, 1.055)	0.892(0.760, 1.047)	0.913(0.777, 1.072)	0.920(0.784, 1.046)
Peer density(KDE)	0.9999813***(0.9999795, 0.9999983)	0.9999865***(0.9999837, 0.9999892)	1.0000053*(0.9999996, 1.0000084)	1.0000144*(0.9999997, 1.0000291)
Edentulous (Y = 2) vs. Limited Functional Capacity (Y = 1)				
Female	0.887***(0.837, 0.940)	0.889***(0.839, 0.942)	0.886***(0.836, 0.939)	0.881***(0.831, 0.943)
Non-Hispanic White	0.935(0.758, 1.153)	0.927(0.750, 1.146)	0.995(0.804, 1.231)	1.042(0.840, 1.149)
Non-Hispanic Black	1.019(0.942, 1.101)	1.018(0.942, 1.099)	1.036(0.961, 1.117)	0.995(0.922, 1.098)
Other non-Hispanic race/ethnicity	0.535*(0.283, 1.012)	0.535*(0.283, 1.013)	0.533*(0.281, 1.013)	0.527*(0.277, 1.016)
Age in years	1.0264***(1.0236, 1.0292)	1.0264***(1.0236, 1.0292)	1.0264***(1.0236, 1.0292)	1.0265***(1.0237, 1.0292)
Former smoker	0.912(0.687, 1.211)	0.911(0.687, 1.209)	0.918(0.691, 1.220)	0.920(0.692, 1.212)
Never smoked	0.813(0.564, 1.173)	0.814(0.564, 1.173)	0.815(0.566, 1.173)	0.823(0.572, 1.172)
Medicaid dental insurance	1.492***(1.299, 1.713)	1.490***(1.297, 1.711)	1.490***(1.297, 1.712)	1.475***(1.285, 1.711)
Private dental insurance	1.430*(0.939, 2.178)	1.427*(0.939, 2.169)	1.436*(0.943, 2.187)	1.434*(0.941, 2.175)
High school education	0.696***(0.603, 0.803)	0.695***(0.602, 0.803)	0.700***(0.606, 0.809)	0.699***(0.604, 0.804)
Collegeeducation	0.481***(0.383, 0.605)	0.480***(0.381, 0.603)	0.488***(0.389, 0.614)	0.492***(0.391, 0.604)
Peer density(KDE)	0.9999990(0.9999972, 1.0000009)	0.9999972***(0.9999950, 0.9999993)	1.0000244*(0.9999995, 1.0000493)	1.0000517*(0.9999947, 1.0001087)
McFadden’s*R* ^2^	0.0644	0.0622	0.0621	0.0633

*** *p* < 0.01; ** *p* < 0.05; * *p* < 0.10

These results provide evidence of systematic differences in oral health among older adults due to peer density, Medicaid coverage, race/ethnicity, smoking status, gender, education, and age. Highlighting the appropriateness of the GACLR model, Medicaid coverage, gender, and age have significant effects on both the odds of limited functional capacity relative to functional dentition and the odds of edentulous relative to limited functional capacity, while peer density (in Model 1), education, smoking status, and race/ethnicity have significant effects on one of these ORs but not the other.

Statistically significant relationships at *p* < 0.01 between peer density (KDE) and improved dentition status, especially for limited functional capacity relative to functional dentition, were found when peer density was measured assuming a more local social network, i.e., with *h* bandwidths of both 0.25 mile (Model 1) and 0.50 mile (Model 2). Because the ORs for the associated KDE variables in Table 2 (upper panel) are less than 1, these peer density effects reduce the likelihood of the negative oral health outcome of limited functional capacity. On the other hand, no significant relationship was found in Model 1 between peer density (KDE) and improved dentition status when comparing edentulous with limited functional capacity (see Table 2, lower panel).

In contrast, peer density effects in Model 2 were statistically significant in reducing the odds of being edentulous given limited functional capacity as well as the odds of developing limited functional capacity given functional dentition. To determine the odds of being edentulous relative to functional dentition, multiply the OR in the upper panel of Table 2 by the corresponding OR in the lower panel. This calculation reveals the OR for Model 1 to be 0.9999803, whereas the OR for Model 2 is 0.9999837. Hence, the combined peer density effect for Model 1 is stronger (further from 1) than that for Model 2. Model 1 also has the greatest goodness of fit, as indicated by the highest McFadden’s pseudo R^2^ among the models tested [41], so it was used to characterize the magnitudes of the estimated effects. Note, though, that the ORs for Models 1 and 2 are essentially the same, especially for those effects that reach statistical significance, so either may have been used.

When comparing the magnitudes of estimated effects, differences in the measurement scale of the variable should be taken into account. For all of the explanatory variables except peer density and age, the OR represents the variable’s total effect given that the variable is binary. For continuous variables such as peer density and age, the OR represents the effect of only a 1-unit change in the variable, so to properly compare the magnitudes of these variables’ effects to those of the binary variables, it is more appropriate to consider the effect of a 1 standard deviation change.

Model 1 reveals that peer density significantly reduces the odds of poor dentition status. Based upon the OR, a 1 standard deviation increase in the density of adults aged 50 years and older per square mile is associated with a 16% reduction in the odds of limited functional capacity relative to functional dentition. While there is no significant effect in Model 1 for edentulous, Model 2 demonstrates that a 1 standard deviation increase in peer density reduces the odds of edentulous relative to limited functional capacity by almost 2% (and the odds of limited functional capacity relative to functional dentition by slightly over 8%). Using the ORs calculated above for edentulous relative to functional dentition, a 1 standard deviation increase in peer density reduces the relative odds of edentulous by roughly 17% in Model 1 and 10% in Model 2 (note that the standard deviation of the peer density measure is a third smaller for *h* = 0.50 than for *h* = 0.25). Another important finding is that participants with Medicaid coverage have 45% higher odds of limited functional capacity relative to functional dentition and 49% higher odds of edentulous relative to limited functional capacity. Combining these 2 effects, the odds of edentulous relative to functional dentition are 116% higher for participants with Medicaid coverage.

The analysis also highlights several interesting sociodemographic differences. Compared to Hispanic participants, the odds of limited functional capacity relative to functional dentition are roughly two-thirds lower for non-Hispanic White, 9% higher for non-Hispanic Black, and almost 41% lower for Other racial/ethnic groups (e.g., Asian Americans). The odds of limited functional capacity relative to functional dentition are also 43% lower for participants who never smoked, while the odds of edentulous relative to limited functional capacity are 30% lower for high school educated and about 52% lower for college educated participants than for those who did not graduate from high school. Not surprisingly, age has similarly strong effects on both ORs. For a 10-year increase in age, participants had 45% higher relative odds of limited functional capacity (upper panel) and almost 30% higher relative odds of being edentulous (lower panel). Combining these 2 effects, a 10-year increase in age is associated with 88% higher odds of edentulous relative to functional dentition. Finally, gender also had strong effects on both ORs but in different directions. Female participants had almost 21% higher relative odds of limited functional capacity and 11% lower relative odds of being edentulous.

Turning to self-rated oral health, Table 3 presents the results of a logistic regression analysis of the likelihood of fair or better vs. poor self-rated oral health.

**Table 3:** Odds ratios (OR) and 95% confidence intervals (CI) for the binary outcome of self-rated oral health (1 = fair or better) resulting from logistic regression with 4 spatial models defined by kernel bandwidth *h*: *ElderSmile* program, New York, NY, 2006-2013 (*n* = 1541 complete cases for self-rated oral health)

	Model 1,*h* = 0.25 mile	Model 2,*h* = 0.5 mile	Model 3,*h* = 1.0 mile	Model 4,*h* = 1.5 mile
Variables	OR (95% CI)	OR (95% CI)	OR (95% CI)	OR (95% CI)
Female	0.908(0.686, 1.202)	0.908(0.686, 1.202)	0.902(0.682, 1.194)	0.902(0.682, 1.202)
Non-Hispanic White	1.074(0.682, 1.668)	1.099(0.706, 1.711)	1.073(0.689, 1.672)	1.068(0.684, 1.716)
Non-Hispanic Black	0.579***(0.427, 0.785)	0.576***(0.426, 0.780)	0.556***(0.412, 0.750)	0.549***(0.406, 0.779)
Other non-Hispanic race/ethnicity	0.724(0.365, 1.437)	0.743(0.374, 1.476)	0.736(0.371, 1.462)	0.730(0.368, 1.475)
Age in years	1.0034(0.9914, 1.0156)	1.0035(0.9915, 1.0157)	1.0037(0.9917, 1.0159)	1.0038(0.9917, 1.0157)
Former smoker	1.139(0.742, 1.747)	1.150(0.750, 1.763)	1.139(0.744, 1.745)	1.136(0.742, 1.761)
Never smoked	1.180(0.779, 1.786)	1.188(0.785, 1.797)	1.192(0.788, 1.803)	1.192(0.788, 1.798)
Medicaid dental insurance	1.346*(0.970, 1.868)	1.338*(0.964, 1.858)	1.340*(0.965, 1.859)	1.338*(0.964, 1.858)
Private dental insurance	1.515(0.874, 2.626)	1.520(0.877, 2.634)	1.497(0.863, 2.597)	1.492(0.860, 2.639)
High school education	1.021(0.743, 1.404)	1.024(0.744, 1.408)	1.014(0.737, 1.394)	1.011(0.736, 1.407)
Collegeeducation	0.783(0.558, 1.098)	0.799(0.569, 1.123)	0.795(0.566, 1.118)	0.792(0.564, 1.123)
Peer density (KDE)	1.0000113**(1.0000008, 1.0000219)	1.0000184*(0.9999978, 1.0000391)	1.0000138(0.9999801, 1.0000475)	1.0000128(0.9999694, 1.0000619)
McFadden’s *R* ^2^	0.0252	0.0254	0.0239	0.0237

*** *p* < 0.01; ** *p* < 0.05; * *p* < 0.10

ORs (and their 95% confidence intervals) are presented in Table 3 for the same 4 model specifications used in the GACLR analysis in Table 2, where the models only differ in the measure of peer density, i.e., which bandwidth was used to construct the kernel density estimate.

As with dentition status, a positive significant association was found between peer density and fair or better self-rated oral health when peer density was measured assuming a more local social network. The estimated effect of peer density was significant at the 5% level in Model 1, when it was measured with a KDE using *h* = 0.25 mile, and at the 10% level in Model 2, when it was measured with a KDE using *h* = 0.50 mile. While Model 2 has slightly better goodness of fit (higher McFadden’s pseudo R^2^), Model 1 is used to characterize the magnitudes of the statistically significant effects, given the stronger statistical significance of the peer density effect and for consistency with the presentation of the dentition status results.

With an OR greater than 1, peer density has a positive effect on the likelihood that a participant self-rates her/his oral health as fair or better. According to Model 1, a 1 standard deviation increase in peer density would increase the odds of a fair or better self-rating of oral health (relative to a poor self-rating) by 11%. It should be noted that Model 2 estimates a similar effect size of just over 12% due to the standard deviation of the peer density measure being a third smaller for h = 0.50. Across all 4 spatial models, Medicaid coverage (*p* < 0.10) and non-Hispanic Black (*p* < 0.01) have significant effects on self-rated oral health. Participants with Medicaid coverage had about 35% higher odds of a fair or better assessment, while participants who identified as non-Hispanic Black were 42% less likely than those who identified as Hispanic to rate their oral health as fair or better.

The results reported in Table 3 validate and extend previous research regarding the effect of spatial peer density on self-rated oral health using *ElderSmile* program data from 2006 to 2009 relative to US Census population data from 2000 [13]. In both studies, a significant (*p* < 0.10) positive association was found for peer density at a bandwidth of *h =* 0.50 mile (Model 2 in Table 3). A new finding of the current study is the emergence of statistical significance (*p* < 0.05) for the peer density effect at a bandwidth of *h* = 0.25 mile (Model 1 in Table 3). Results of the current study are consistent with the previous study’s findings of racial/ethnic differences in and the positive effect of Medicaid coverage on self-rated oral health, while differing in not finding a significant negative effect for women. The models in the current study, though, more appropriately account for racial/ethnic differences and control for other risk factors, thereby increasing confidence in the estimated effect sizes reported here. Another strength of the current results is that the models were estimated with more information, due to both the use of multiple imputation to retain records with missing values and the larger number of observations in the 2006-2013 dataset than in the 2006-2009 dataset.

Finally, comparing the results in Tables 2 and 3 provides insight on how peer density and the sociodemographic predictors in the model influence the 2 measures of oral health. Peer density had consistently positive effects on dentition status and self-rated oral health. In contrast, participants who identified as non-Hispanic Black had lower odds of a fair or better self-rating of their oral health and higher odds of limited functional capacity relative to functional dentition. Other predictors had a strong effect on dentition status but a small or statistically insignificant effect on self-rated oral health. Medicaid coverage and age increased the relative odds of poorer dentition status (edentulous relative to limited functional capacity and limited functional capacity relative to functional dentition) but had a positive and null effect, respectively, on the likelihood of a fair or better self-assessment. Similarly, having never smoked and earning more than a high school education reduced the relative odds of a poorer dentition outcome, whereas their effects on self-rated oral health were insignificant. These seemingly inconsistent findings may be due to the fact that older adults with Medicaid coverage may have their teeth pulled when they seek dental care because Medicaid fails to cover more extensive procedures such as root canals that may preserve the dentition. Nonetheless, the treatment (loss) of infected teeth may result in less pain and hence better self-rated oral health. Another possibility is that Medicaid coverage is a surrogate for other potential covariates omitted from the model due to data limitations, such as economic means. The distinctness of these 2 outcomes is also reflected by the findings that language and private dental insurance have no significant effects on dentition status, while accounting for significant differences in self-rated oral health.

## Discussion

This study provides novel and compelling evidence of the relationship between peer density and oral health for older adults that extends previous exploratory research using self-rated oral health as the outcome [7]. Results from the GACLR analysis of dentition status, as derived from the number of missing teeth, demonstrates that the relationship between peer density and oral health for older adults is not limited to a single oral health outcome. Moreover, a methodological advance of this study is the use of multiple imputation as a strategy for dealing with missing data among explanatory variables to make statistical inferences regarding oral health outcomes of interest [21].

The relatively small bandwidths exhibiting statistical significance for dentition status in this study, namely *h* = 0.25 mile (Model 1) and *h* = 0.50 mile (Model 2), are consistent with the dense urban environment of northern Manhattan and the Bronx, where many opportunities and activities are concentrated in neighborhoods within walking distance from home. Therefore, using smaller bandwidths better characterizes peer density effects than larger bandwidths for this study area and older adult population. Indeed, while a comparison of the 4 models for dentition status in Table 2 demonstrates the robustness of the findings for the other predictors of dentition status, Models 1 and 2 arguably employ the most appropriate peer density measures, given the geographic characteristics of Manhattan and the Bronx, the significant variation of the older adult population density across Census blocks, and the theoretical expectation that the social networks of older adults become more geographically compact with older age.

Determining the appropriate kernel bandwidth *h* at which to represent peer density involves a trade-off between the uncertainty and the bias of the estimated density relative to the true density at a given point. Because a larger bandwidth includes more observations, it reduces the uncertainty of the estimate, but also induces a bias toward less spatial variation between points. Because a smaller bandwidth induces more spatial variation between points, the bias of the estimate is reduced but it carries greater uncertainty due to fewer observations being used in the calculation. The kernel density surface created for Model 2 at *h* = 0.50 mile may provide a useful balancing of this trade-off between uncertainty and bias, as it smooths over the variation apparent at the smallest bandwidth (*h* = 0.25 mile) surface (Model 1), but exhibits clustering more than the surfaces used for Model 3 and Model 4 at larger bandwidths. This balancing of uncertainty and bias may contribute to the finding that Model 2 at the *h* = 0.50 mile bandwidth is the most statistically significant regarding self-rated oral health.

Taken together, these results indicate that models using smaller *h* bandwidths have a better fit and thus underscore the potential importance of the local social context in promoting oral health for older adults. This finding is consistent with a previous report that found social network density and proximity to other older adults has a positive significant association with perceived social connectedness, which in turn has a positive significant association with health status [42]. Likewise, an earlier study documented the adverse effects of social disconnectedness and perceived isolation on the health of older adults [43].

### Limitations

This research has certain limitations. Since *ElderSmile* participants are community-based older adults who elected to partake in the program activities, insights from this study reflect the relatively mobile and social older adult population living in the disadvantaged neighborhoods of northern Manhattan and the Bronx, rather than the older adult population as a whole. There is thus a potential for selection bias in the findings that limits their generalizability. In particular, these peer density findings might not hold as strongly for the entire population of older adults, who likely have less dense social networks composed of weaker ties relative to the study participants. Further, the results are less generalizable to rural and suburban settings than they are to other dense urban areas with geographically small Census blocks. Future research on peer density effects ought to consider the relevant urban geographies when choosing how best to measure the concept, as areas with less walkable environments or lower population density may warrant the use of larger bandwidths to adequately represent the local social context. In extending this approach to other geographical contexts, including a range of bandwidths (as in the present study) is advisable to facilitate determination of an appropriate kernel density surface for representing a context-specific peer density effect.

While the results of this study confirm an earlier report of an association between peer density and oral health [13], more research is needed to fully explicate the causal mechanisms underlying this relationship. Further investigation is also needed to confirm and explicate the associations revealed between oral health and Medicaid coverage, race/ethnicity, gender, smoking, and education. Finally, it may also be useful to consider whether the effects of peer density vary with race/ethnicity and education. As such, this study informs ongoing research by the study team that seeks to explore these mechanisms using dynamic modeling and simulation of social networks to investigate their potential influence on oral health equity.

## Conclusions

This study provides new evidence that the oral health of community-based older adults is affected by peer density in an urban environment. In exploring relationships between peer density and 2 measures of oral health (dentition status and self-rated oral health), this research demonstrates that peer density surfaces constructed from publicly available US Census data may be useful for characterizing the social context of older adults in urban areas. To the extent that peer density signifies the potential for social interaction and support, the positive significant effects of peer density on improved oral health point to the importance of place in promoting social interaction as a component of healthy aging. While different racial/ethnic peer density effects on physical morbidity, mortality, and health behaviors have been postulated and contested [44], this study underscores that the local social context as measured by peer age-group density may play an important role in promoting health for older adults. Recognizing this role, policies that promote aging in place might leverage the proximity of peers and their knowledge of the local context to promote oral and general health.

## Abbreviations

CI: confidence interval
GACLR: generalized adjacent categories logistic regression
KDE: kernel density estimate
OR: odds ratio
VGAM: vector generalized linear and additive models

## References

[1] Lamster IB (2004). Oral health care services for older adults: a looming crisis. Am J Public Health.

[2] Centers for Disease Control and Prevention (2013). The state of aging and health in America 2013.

[3] Amaro H (2014). The action is upstream: place-based approaches for achieving population health and health equity. Am J Public Health.

[4] Smedley B, Amaro H (2016). Advancing the science and practice of place-based intervention. Am J Public Health.

[5] Tsakos G, Demakakos P, Breeze E, Watt RG (2011). Social gradients in oral health in older adults; findings from the English longitudinal survey of aging. Am J Public Health.

[6] Griffin SO, Jones JA, Brunson D, Griffin PM, Bailey WD (2012). Burden of oral disease among older adults and implications for public health priorities. Am J Public Health.

[7] Marshall SE, Cheng B, Northridge ME, Kunzel C, Huang C, Lamster IB (2013). Integrating oral and general health screening at senior centers for minority elders. Am J Public Health.

[8] Northridge ME, Kum SS, Chakraborty B (2016). Third places for health promotion with older adults: using the consolidated framework for implementation research to enhance program implementation and evaluation. J Urban Health.

[9] Petersen PE, Kwan S (2004). Evaluation of community-based oral health promotion and oral disease prevention – WHO recommendations for improved evidence in public health practice. Community Dent Health.

[10] Campus G, Salem A, Uzzau S, Baldoni E, Tonolo G (2005). Diabetes and periodontal disease: a case-control study. J Periodontol.

[11] Minassian C, D’Aiuto F, Hingorani AD, Smeeth L (2010). Invasive dental treatment and risk for vascular events: a self-controlled case series. Ann Intern Med.

[12] Bosworth HB, Schaie KW (1997). The relationship of social environment, social networks, and health outcomes in the Seattle longitudinal study: two analytical approaches. J Gerontol B Psychol Sci Soc Sci.

[13] Widener MJ, Metcalf SS, Northridge ME, Chakraborty B, Marshall SM, Lamster IB (2012). Exploring the role of peer density in the self-reported oral health outcomes of older adults: a kernel density based approach. Health Place..

[14] Metcalf SS, Northridge ME, Widener MJ, Chakraborty B, Marshall SE, Lamster IB (2013). Modeling social dimensions of oral health among older adults in urban environments. Health Educ Behav.

[15] Kirmeyer SL (1978). Urban density and pathology: a review of research. Environ Behav.

[16] Kennedy-Hendricks A, Schwartz HL, Griffin BA (2015). Health implications of social networks for children living in public housing. Health Place.

[17] Uchino BN (2006). Social support and health: a review of physiological processes potentially underlying links to disease outcomes. J Behav Med.

[18] Evans GW, Lercher P, Kofler WW (2002). Crowding and children's mental health: the role of house type. J Environ Psych.

[19] McLafferty S, Widener M, Chakrabarti R, Grady S (2012). Ethnic density and maternal and infant health inequalities: Bangladeshi immigrant women in new York City in the 1990s. Ann Assoc Am Geogr.

[20] US Census Bureau. 2010 Census Data. http://www.census.gov/2010census/data/. Accessed 12 Jul 2017.

[21] Rubin DB (1978). Multiple imputations in sample surveys--a phenomenological Bayesian approach to nonresponse. Proceedings of the survey research methods section of the American Statistical Association.

[22] Lamster IB, Northridge ME (2008). Improving oral health for the elderly: an interdisciplinary approach.

[23] Marshall S, Northridge ME, De La Cruz LD, Vaughan RD, O'Neil-Dunne J, Lamster IB (2009). ElderSmile: a comprehensive approach to improving oral health for seniors. Am J Public Health.

[24] Northridge ME, Ue FV, Borrell LN (2012). Tooth loss and dental caries in community-dwelling older adults in northern Manhattan. Gerodontology.

[25] Northridge ME, Chakraborty B, Kunzel C, Metcalf S, Marshall S, Lamster IB (2012). What contributes to self-rated oral health among community-dwelling older adults? Findings from the ElderSmile program. J Public Health Dent.

[26] Northridge ME, Yu C, Chakraborty B (2015). A community-based oral public health approach to promote health equity. Am J Public Health.

[27] Silverman BW (1986). Density estimation for statistics and data analysis.

[28] Zia A, Norton BG, Metcalf SS, Hirsch PD, Hannon BM (2014). Spatial discounting, place attachment, and environmental concern: toward an ambit-based theory of sense of place. J Env Psych.

[29] Dye BA, Tan S, Smith V (2007). Trends in oral health status: United States, 1988-1994 and 1999-2004. Vital Health Stat 11.

[30] World Health Organization (1992). Recent advances in health. WHO technical report series no. 826.

[31] Carpenter JR, Kenward MG (2013). Multiple imputation and its application.

[32] Arnold AM, Kronmal RA (2003). Multiple imputation of baseline data in the cardiovascular health study. Am J Epidemiol.

[33] McCaul KA, Almeida OP, Norman PE (2015). How many older people are frail? Using multiple imputation to investigate frailty in the population. J Am Med Dir Assoc.

[34] Rubin DB (1976). Inference and missing data. Biometrika.

[35] Schafer JL (1997). Analysis of incomplete multivariate data. Monographs on statistics and applied probability.

[36] Zhang P (2003). Multiple imputation: theory and method. Int Stat Rev.

[37] Little RJ (1988). Missing-data adjustments in large surveys. J Bus Econ Stat.

[38] Brand JPL, Buuren S, Groothuis-Oudshoorn K, Gelsema ES (2003). A toolkit in SAS for the evaluation of multiple imputation methods. Statistica Neerlandica.

[39] King G, Honaker J, Joseph A, Scheve K (2001). Analyzing incomplete political science data: an alternative algorithm for multiple imputation. Am Polit Sci Rev.

[40] Agresti A (2010). Analysis of ordinal categorical data.

[41] McFadden D, Zarembka P (1974). Conditional logit analysis of qualitative choice behavior. Frontiers in economics.

[42] Ashida S, Heaney CA (2008). Differential associations of social support and social connectedness with structural features of social networks and the health status of older adults. J Aging Health.

[43] Cornwell EY, Waite LJ (2009). Social disconnectedness, perceived isolation, and health among older adults. J Health Soc Behav.

[44] Becares L, Shaw R, Nazoo J (2013). Ethnic density effects on physical morbidity, mortality, and health behaviors: a systematic review of the literature. Am J Public Health.

